# Cognitive Ability and the Demand for Redistribution

**DOI:** 10.1371/journal.pone.0109955

**Published:** 2014-10-24

**Authors:** Johanna Mollerstrom, David Seim

**Affiliations:** 1 Interdisciplinary Center for Economic Science, and Department of Economics, George Mason University, Fairfax, Virginia, United States of America; 2 Program for Evolutionary Dynamics, Harvard University, Cambridge, Massachusetts, United States of America; 3 Department of Economics, University of Toronto, Toronto, Ontario, Canada; 4 Research Institute of Industrial Economics, Stockholm, Sweden; University of Vienna, Austria

## Abstract

Empirical research suggests that the cognitively able are politically more influential than the less able, by being more likely to vote and to assume leadership positions. This study asks whether this pattern matters for public policy by investigating what role a person's cognitive ability plays in determining his preferences for redistribution of income among citizens in society. To answer this question, we use a unique Swedish data set that matches responses to a tailor-made questionnaire to administrative tax records and to military enlistment records for men, with the latter containing a measure of cognitive ability. On a scale of 0 to 100 percent redistribution, a one-standard-deviation increase in cognitive ability reduces the willingness to redistribute by 5 percentage points, or by the same amount as a $35,000 increase in mean annual income. We find support for two channels mediating this economically strong and statistically significant relation. First, higher ability is associated with higher income. Second, ability is positively correlated with the view that economic success is the result of effort, rather than luck. Both these factors are, in turn, related to lower demand for redistribution.

## Introduction

Prior research suggests that the cognitively able are politically more influential than the less able, by being more likely to vote [1] and to assume leadership positions [1], [2]. If political preferences vary by ability, implemented policies may therefore be skewed in favor of the cognitively able. Although intelligence indeed is negatively correlated with general attitudes such as expressing racist opinions [3]–[5], or prejudice towards homosexuals [3], [6] there is little work on the relation between cognitive ability and preferences over actual policies. In this study, we ask whether individuals of higher cognitive ability demand more or less redistribution of income between citizens in society, compared to those of lower ability. This question is important since most governments redistribute income, and policies with redistributive components, e.g. social security, government transfers and progressive tax schemes, have increased vastly in importance during the last decade [7].

Preferences for redistribution are the focus of a large academic literature. The canonical view that individuals are self-interested and demand less redistribution as their income and wealth increase has found empirical support [8], [9], but has also been scrutinized and challenged. For instance, locus of control in the form of beliefs about the extent to which individuals' economic outcomes can be attributed to effort rather than to luck has been shown to be a stronger determinant of preferences for redistribution than income [10], [11]. Moreover, if the income process is thought to be dominated by factors beyond the individual's control, redistribution provides insurance as it increases disposable income for those members of society whose income is low. With a greater aversion towards risk, this role becomes more prominent and demand for redistribution increases [12]. However, intervening in the economy creates distortions, and those who believe that these policies will lead to a reduction in the size of the economic pie are less likely to be supporters of redistribution [13]–[15]. Finally, individual preferences such as altruism have been shown to be important for the demand for redistribution [15], [16].

In this paper, we analyze the relation between cognitive ability and demand for redistribution. We start by documenting the statistical correlation between the two, an association that has not been assessed previously. It is, however, also important to disentangle the underlying mechanisms behind the overall effect. Previous research shows that the factors discussed above are, in addition to being important for the demand for redistribution, also covarying with cognitive ability. For example, cognitive ability has been argued and shown to be an important determinant of both labor market success and socio-economic status [17], [18]. From a self-interest point of view, able individuals could thus be expected to demand lower redistribution because they have higher incomes. This is especially true if they believe income and socio-economic status to be the result of effort and hard work rather than of luck. Moreover, the documented higher willingness to take on risk among the cognitively able [19]–[22], may lead them to demand less redistribution, even if cognitive ability were not associated with income or beliefs about the income-generating process. On the other hand, high-ability individuals tend to be more altruistic [23], [24], which could imply a higher demand for redistribution. Also, beliefs regarding the distortionary effects of redistribution may also vary with ability. We use mediation analysis to decompose the aggregate effect of cognitive ability on preferences for redistribution into underlying mechanisms [25]–[27]. Based on the previous literature, we test the following mediators: 1) Income, 2) beliefs about the determinants of economic success, 3) beliefs about how efficiently the public sector redistributes resources, 4) altruism, measured as willingness to donate to charitable purposes, and 5) risk aversion.

We employ several data sets, all linked at the individual level. We elicit preferences for redistribution using a carefully constructed questionnaire. While previous studies resort to existing surveys eliciting general opinions about inequality and government interventions, our questionnaire defines, more precisely, redistribution as meaning that “the public sector, through taxes and subsidies, makes income in society more equal between the citizens than what would have been the case without those taxes and subsidies.” We explicitly ask individuals to indicate their preferred level of income redistribution on a scale that ranges from no redistribution (defined in the questionnaire as meaning that “the public sector does not influence the income distribution at all”) to full redistribution (“everyone receives the same income after taxes and subsidies”).

The questionnaire also elicits beliefs about whether luck or effort determines economic success and about whether the public sector is efficient in redistributing income between citizens. In addition, the survey measures altruistic preferences (in the form of willingness to give to charitable purposes) and risk aversion. For more details on the questionnaire, see the Methods section below.

Next, we match the questionnaire responses to individual military enlistment records, comprising a measure of cognitive ability. The cognitive ability test used at military enlistment in Sweden consists of four subtests (logical ability, verbal ability, technological comprehension and metal folding). The results of these tests are transformed to a single measure, which is a recognized measure of intelligence [17], [18], [28]. Military enlistment at age 18 was mandatory for men in our sample, in contrast to the AFQT test in the USA, which is voluntary. It is also important to note that it was not possible to avoid military service with a low score on the test. [18]

Self-reported income and wealth data are plagued by measurement errors due to, for example, imperfect recall. The problem is arguably particularly severe when asking subjects about comprehensive income and wealth histories. Such accurate trajectories constitute proxies for lifetime income and wealth that are likely to matter as much for the demand for redistribution as current, transitory, income. Hence, when analyzing the mediating role of income and wealth for the relationship between cognitive ability and the demand for redistribution, inaccurate measures may obscure important relationships and make these roles difficult to assess. To address these issues, we link the matched survey and enlistment data to administrative tax records at the individual level. This gives us accurate annual labor and capital income histories for twelve years prior to the collection of the survey data. Moreover, Sweden taxed wealth until 2007 and we are able to retrieve records of individuals' net worth. Taken together, these data provide high-quality proxies for lifetime income and wealth. In addition, the administrative data include demographic data on education and government transfers.

The survey was distributed by mail to a representative sample of Swedish men above 18 years of age in May 2011. The response rate was 36 percent and our sample consists of 271 men.

Our approach of using the answers from a questionnaire designed specifically to elicit redistributional preferences, linked to military enlistment data on cognitive ability and administrative data on earnings, wealth, transfers and demographics provides a unique setting for analyzing the role of cognitive ability in determining preferences for redistribution. We relate the respondents' cognitive ability at age 18 to their demand for redistribution at ages 33–61 and explore different channels through which ability might influence this relation.

## Results

**Figure 1:**
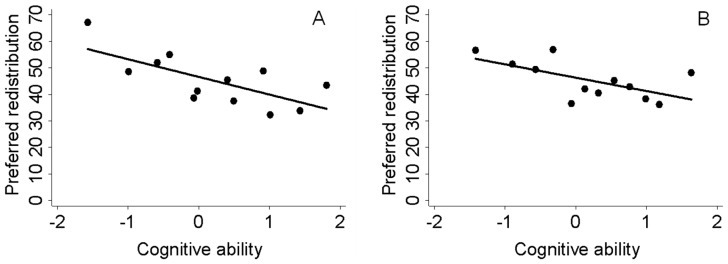
Cognitive ability and demand for redistribution. Visual representation of the relation between cognitive ability and demand for redistribution. Preferred redistribution defined as in text, ranging from no (0 percent) to full (100 percent) redistribution. Cognitive ability scaled to have mean 0 and sd 1 in the sample of all enlisters. We rank individuals according to cognitive ability and construct twelve equal-sized bins. The figures show mean redistribution against mean cognitive ability in each bin. N = 271. Panel A: Raw correlation. Panel B: Controlling for age, for whether subject continued from primary to secondary school, and for socio-economic status during childhood (answer to question “How would you classify yourself in terms of class when you grew up?” with alternatives “Working class”, “Lower middle class”, “Middle class”, “Upper middle class”, “Upper class”). To obtain this figure we first regress demand for redistribution on these control variables. We then add the mean of the demand for redistribution variable to the residuals obtained from that regression and plot this variable against cognitive ability.

Our results show that individuals with higher cognitive ability demand less redistribution. Plotting demand for redistribution, measured on a scale from 0 (no redistribution) to 100 (full redistribution) against our standardized measure of cognitive ability, Panel A of Figure 1 illustrates this negative and linear relation. Regressing preferences on cognitive ability using Ordinary Least Squares (OLS) in specification 1 in Table 1 reveals that a one-standard-deviation increase in cognitive ability is associated with a 6.7 percentage point lower demand for redistribution (or, equivalently, a 15 percent decrease in mean willingness to redistribute). The magnitude drops to 5.0 percentage points when we control for age, self-reported socio-economic status during childhood and whether the individual proceeded from primary to secondary education (Panel B of Figure 1 and specification 2 in Table 1). By including these controls we alleviate the concern that our measure of cognitive ability could be mainly capturing general childhood characteristics and educational differences prior to taking the ability test, rather than ability in itself. All results hold if Ordered Probit (OP) is used instead of OLS (see Table S1 in File S1).

**Table 1:** Demand for redistribution, regression analysis.

	(1)	(2)	(3)	(4)	(5)	(6)	(7)	(8)	(9)	(10)	(11)	(12)	(13)	(14)	(15)	(16)
Cognitive ability	−6.665***	−5.026***		−4.264**		−4.238**		−4.819***		−4.415**		−4.846***		−3.696**		−3.475*
	(1.621)	(1.723)		(1.769)		(1.759)		(1.683)		(1.778)		(1.749)		(1.796)		(1.858)
																
Mean annual income			−0.235**	−0.178**									−0.138	−0.138	−0.139	−0.101
			(0.092)	(0.089)									(0.087)	(0.087)	(0.099)	(0.095)
																
Beliefs: luck/effort					−2.874***	−2.577***							−2.403**	−2.403**	−2.247**	−2.146**
					(0.990)	(0.985)							(0.992)	(0.992)	(1.051)	(1.048)
																
Beliefs: gov efficiency							2.188***	2.176***							2.231***	2.183***
							(0.747)	(0.751)							(0.750)	(0.751)
																
Risk aversion									0.876	0.911					0.661	0.736
									(0.896)	(0.877)					(0.877)	(0.864)
																
Altruism											−0.151	0.111			−0.097	−0.005
											(0.723)	(0.723)			(0.676)	(0.680)
																
Standard controls	No	Yes	Yes	Yes	Yes	Yes	Yes	Yes	Yes	Yes	Yes	Yes	Yes	Yes	Yes	Yes
N	271	266	266	266	265	265	262	262	247	247	262	262	265	265	243	243

OLS. Robust standard errors in parentheses. Significance levels: ***p<0.01, **p<0.05, *p<0.1. Dependent variable is demand for redistribution measured in percent where 0% =  no redistribution, 100% = full redistribution. Cognitive ability measure from military enlistment data with zero mean and unit standard deviation in the population of all enlisters. Standard controls are age, education level (dummy for primary school being highest education level), and self-reported socio-economic status during childhood (alternatives "working class", "lower middle class", "middle class", "upper middle class" and "upper class"). Mean annual income is measured in SEK 10,000 s, mean taken over the years 1999 to 2010. Beliefs about luck/effort measured with question in survey about what matters (luck or effort) for how well an individual does economically in life, higher number indicates more importance for effort. Beliefs about government efficiency measured in survey with question about how efficiently the public sector in Sweden redistributes resources so that no resources get lost on the way, higher number indicate more efficiency. Risk aversion measured in survey with eight hypothetical choices between a fixed amount and a lottery, risk aversion is sum of choices of the fixed amount. Altruism assessed in survey with hypothetical question about the willingness to give to charitable purposes (1 = willing to give, 0 = not willing to give). All results are robust to using Ordered Probit (OP) instead of OLS (results available in Table S1 in SI).

The effect of cognitive ability on the demand for redistribution is large: to change the demand by as much as a one-standard-deviation increase in cognitive ability does, an individual has to experience an increase in mean annual income over the last twelve years of about $35,000 (or 1.25 standard deviations). To see this, first consider specification (3) in Table 1 which reveals that the coefficient of mean annual income, measured in SEK 10,000 s, in a regression where demand for redistribution is the dependent variable amounts to −0.235. Multiplying this by 6.1 (the exchange rate of USD to SEK at the time of the questionnaire) gives us the effect that an increase in mean annual income of USD 10,000 has on demand for redistribution.

Investigating potential mechanisms for this result, we find a positive relation between cognitive ability and mean annual income: Controlling for childhood demographics, a one-standard-deviation increase in cognitive ability increases mean annual income by about $7,000 (SEK 42,700, p<0.01, see Table 2). The demand for redistribution is, in turn, negatively associated with mean annual income (b = −0.24, p<0.05, see specification 3, Table 1). The effect of cognitive ability on the demand for redistribution remains significant but is slightly smaller when we control for mean annual income (specification 4, Table 1). On its own, mean annual income is a marginally statistically significant, partial mediator of the relation between cognitive ability and demand for redistribution. A test of mediation [25], [26] reveals that the point estimate of the indirect effect of cognitive ability influencing the demand for redistribution through mean annual income amounts to −0.762 (p = 0.099), and the proportion of the effect of ability that is mediated is 16 percent.

**Table 2:** Correlates of cognitive ability.

	Mean annual income	Beliefs: luck/effort	Beliefs: gov efficiency	Risk aversion	Altruism
Cognitive ability	4.269***	0.294**	−0.015	0.045	0.359**
	(1.070)	(0.125)	(0.145)	(0.152)	(0.158)
Standard controls	Yes	Yes	Yes	Yes	Yes
N	266	265	262	247	262

Coefficient on cognitive ability from an OLS regression where the variable indicated in the column is the dependent variable. Significance levels: ***p<0.01, **p<0.05, *p<0.1. The regressions include standard controls. These are age, education level (dummy for primary school being highest education level), and self-reported socio-economic status during childhood (alternatives "working class", "lower middle class", "middle class", "upper middle class" and "upper class"). Robust standard errors in parentheses. All variables are defined as in table 1.

Our test of mediation is a version of Sobel [25], [26]. For both the simple mediation models with one mediation variable, and for multiple mediation which is discussed further below, we start by simultaneously running the regression(s) where the mediating variables are explained by cognitive ability and the standard set of controls, together with the full regression where redistributional preferences are explained by the mediator(s), cognitive ability, and control variables. In the estimations, we do not assume that the error terms are uncorrelated. We apply the delta method to convert the individual standard errors to produce the standard error for the indirect effect [27]. The mediation results reported here are robust to using non-parametric bootstrap methods, resampling the observations 1000 times.

For expositional convenience, only mean annual income is used in the regressions presented in Table 1. However, our conclusions from Table 1 are robust to using other proxies for life-time income. In Table S2 in File S1 we show that the results obtain also when we (i) include only current income and allow for nonlinear income effects; (ii) allow for nonlinear income effects in mean annual income; (iii) control for source of income (capital or labor); (iv) include measures of income variability; (v) include government transfers; (vi) include individual beliefs about future relative income; (vii) control for wealth (and possible non-linear effects of it); and (viii) control for source of wealth (financial or non-financial). Moreover, Table S3 in File S1 shows that the effect of cognitive ability on the demand for redistribution is not weaker for older individuals (which we would expect if our results were impacted by income mismeasurement, as life-time income is more correctly measured for older individuals).

Turning to differences in beliefs about the income-generating process, specification 5 in Table 1 shows that the belief that economic success is the result of effort rather than luck is negatively correlated with the demand for redistribution (b = −2.87, p<0.01). This is true also when including cognitive ability in the estimation (specification 6 in Table 1). Furthermore, our results indicate that high-ability individuals believe income to be determined by effort to a larger extent than low-ability individuals. (b = 0.294, p<0.05, see Table 2). The test of mediation reveals that the point estimate of the indirect effect of cognitive ability influencing the demand for redistribution through this channel is −0.758 (p = 0.062) and that the proportion of the effect of ability that is mediated is 15 percent. Hence, beliefs about luck or effort being the main determinant behind economic success is on its own a marginally statistically significant partial mediator of the relation between cognitive ability and demand for redistribution.

The belief that the government redistributes efficiently is positively and significantly correlated with support for redistribution (see specifications 7 and 8 in Table 1). However, these beliefs have no statistically significant relation with cognitive ability (see Table 2) and this channel is no mediator in the composite relation between ability and preferences (indirect effect of −0.032, p = 0.92). The same is true also for risk aversion (cf. specification 9 and 10 in Table 1, and Table 2, indirect effect of 0.041, p = 0.77). Last, we show, in line with previous research, that the more cognitively able, despite being less willing to redistribute, are *more* prone to express altruistic preferences in the form of willingness to donate to charitable organizations (b = 0.36, p<0.05, see Table 2). It is therefore not surprising that the test of mediation confirms that altruism is not mediating the negative relation between cognitive ability and demand for redistribution (indirect effect of 0.040, p = 0.87, see also specification 11 and 12 in Table 1).

**Figure 2:**
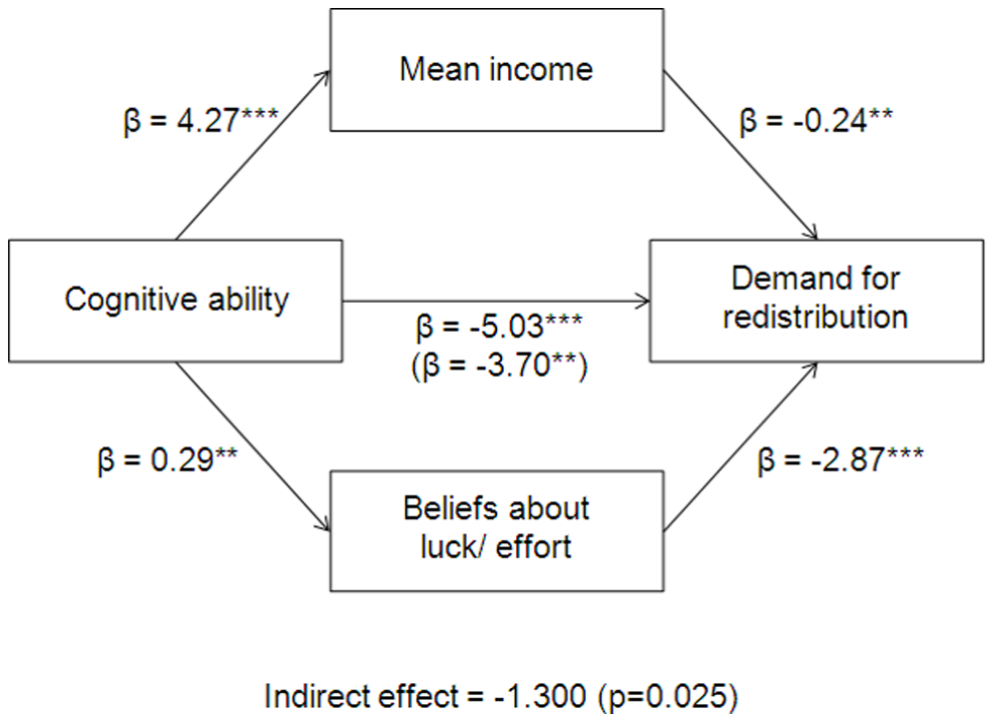
Income and beliefs about the importance of luck/effort for economic success are significant partial meditators. All regressions in the mediation analysis include standard controls. Significance levels: *** p<0.01, ** p<0.05, * p<0.1. All variables are defined as in Table 1. All coefficients reported in this figure can also be found in Tables 1 and 2, except for β = −3.70**. This is the coefficient on cognitive ability from an OLS regression where demand for redistribution is the dependent variable and mean annual income *and* beliefs about the luck/effort are included in addition to the standard controls.

We conclude that mean annual income and beliefs about the extent to which effort rather than luck is the determinant of economic success are partial mediators in the relation between cognitive ability and the demand for redistribution. We run a model of multiple mediators, depicted in Figure 2, with these two channels in order to understand the proportion of the relation that they can explain together. These two statistically significant mediators of the relation between cognitive ability and demand for redistribution, with a joint point estimate is −1.300 (p = 0.025), account for 26 percent of the effect of cognitive ability on preferences for redistribution. The fact that these factors together have an explanatory power which is almost twice as strong as either of them individually also confirms that the two channels capture different aspects of the relation between cognitive ability and demand for redistribution.

## Discussion

In this study we found clear evidence of an economically large and statistically significant negative association between individuals' cognitive ability and their views on the redistribution of income in society. This finding is important because high-ability individuals are more prone to being politically active, suggesting that policies may be different than what would have been the case if the views of the full population had been equally reflected.

Analyzing an array of potential channels, we find that mean annual income and beliefs about the income-generating process are partial mediators in this relation.

The empirical approach uses unique data from a questionnaire designed specifically to elicit preferences for redistribution, administrative tax records comprising income and wealth trajectories as well as enlistment data that include a measure of cognitive ability, all linked at the individual level. Since the ability variable was collected at age 18 and because we have information about childhood characteristics, the effect of cognitive ability will not simply represent growing up in environments with high or low demand for redistribution or having more or less education before age 18.

We also find that cognitive ability is associated with the belief that income is determined by effort rather than luck, and that this relation is a significant mediator of the composite relation between ability and the demand for redistribution. This may have implications beyond the particular example of economic policy studied here, not least for policies related to social mobility. This study is one step towards a deeper understanding of in what ways cognitive ability influences policy. As Sweden has a relatively compressed income distribution, and as our sample is comparatively small and consists of men only, we look forward to future research that considers larger samples including also women, and that uses data from other countries.

## Method

The data set we use consists of survey responses, military enlistment records and administrative records. The survey material was collected by Statistics Sweden, who linked the data with demographic information and income histories on the individual level using the data base LISA and the National Service Administration. Since cognitive ability data come from military enlistment, our sample consists of men only (see further discussion below). Summary statistics are shown in Table 3.

**Table 3:** Summary statistics.

Variable	Mean	Sd	Median	Min	Max	N
Cognitive ability	0.18	0.98	0.03	−2.19	2.09	271
Demand for redistribution	45.4	26.1	44.4	0	100	271
Age	47.2	8.4	48	33	61	271
Mean annual income	315 557	170 804	286 914	15 256	1 346 648	271
Primary school	0.12	0.33	0	0	1	271
Secondary school	0.47	0.5	0	0	1	271
Tertiary school	0.41	0.49	0	0	1	271
Luck/effort	6.3	1.8	7	1	10	269
Government efficiency	3.6	2.15	3	1	10	265
Altruism	4.6	2.4	5	1	10	266
Risk aversion	4.9	2.0	5	0	8	249

All variables defined as in Table 1 except for Mean annual income which is here in SEK. Note that Demand for redistribution was measured on a 1–10 scale in the survey. In the analysis we rescaled the variable to range from 0 to 100 so that it can be interpreted as percentage redistribution desired. The median value of 44.4 hence corresponds to a choice of 4 on the original scale.

The survey was distributed by mail to a representative sample of Swedish men above 18 years of age in May 2011, with a response rate of 36 percent (similar to other mail surveys conducted by Statistics Sweden). To assess the possibility of non-random attrition, we compare means within the responding sample to those within the full population of Swedish men. Using the extensive administrative data to investigate variables that are believed to influence redistributive preferences, such as the value of real estate, financial net wealth, number of children, labor earnings, the indicator of having divorced, and having obtained secondary education, we find that mean values are very similar.

### Questionnaire

The questionnaire was designed by us and implemented by Statistics Sweden. Subjects were asked to indicate their preferred level of income redistribution on a scale ranging from no to full redistribution. The scale was presented to subjects with 10 steps, with 1 being defined as no redistribution (meaning “the public sector does not influence the income distribution at all“) and 10 as full redistribution (“everyone receives the same income after taxes and subsidies”). In the analysis, we rescale the variable to range from 0 to 100 and it can therefore be interpreted as percent redistribution desired.

Respondents' beliefs about whether effort or luck matter for economic success was elicited with the following question: “Is it mostly individual effort or luck that matters for how well an individual does economically in life?” Respondents indicated their opinion on a scale from 1 (defined as “only luck”) to 10 (“only individual effort”). Beliefs about how efficiently the public sector redistributes resources were captured by the question: “To what extent do you agree with the following statement: ‘The public sector is efficient when redistributing money (no money is lost on the way)’”. The answer was given on a scale from 1 (defined as “I disagree completely”) to 10 (“I agree completely”).

To measure risk preferences our survey utilizes a set of eight questions where the respondent makes hypothetical choices between receiving a fixed amount of money and participating in a lottery. Whereas the lottery remains the same in all eight questions, the fixed amount varies from 1/3 to 5/3 of the expected value of the lottery (the exact question posed was: “Below we ask you a few questions where you can choose between getting a fixed sum of money for sure or to take part in a lottery where you have a 50% chance of winning 3000 SEK (492 USD) and a 50% chance of not winning anything. We vary the alternative that you can get for sure but the lottery stays the same. Please note that all choices are hypothetical.”). The number of times a participant chooses the fixed amount is a standard measure of risk aversion [29], [30]. We elicited a measure of one aspect of altruistic preferences in the survey with a question about the willingness to give money to charitable organizations: “If you would win SEK 10 000 [1639 USD] in a lottery, would you give anything to a charity?” Both this measure and the risk preference measure have been shown to yield similar results when incentivized and when used in a context without monetary incentives, such as a survey [30].

### Cognitive ability measure

Military enlistment takes place in the year a Swedish man turns 18 or 19. The cognitive ability test, a mandatory part of the enlistment procedure, consists of four sub-tests (logical ability, verbal ability, technological comprehension and metal folding) with 40 questions each. The results of these tests are transformed to a cognitive skills variable that ranges from one to nine. We standardize this variable so that it has zero mean and unit variance in the population of all Swedish men born between 1951 and 1979. The cognitive ability measure is considered consistent between the years of 1969 and 1997 [18], [19] and we exclude (before conducting any analysis) the men in our sample who enlisted before 1969 or after 1997. Since military enlistment was never mandatory for Swedish women we exclude, before conducting any analysis, the very few women for whom data are available. This strategy is in line with previous studies using these data [17], [18], [28].

### Administrative data

The source of administrative data is the longitudinal integration database for health insurance and labor market studies (LISA by Swedish acronym). LISA contains individual information on taxable labor and capital income, financial and other wealth, government transfers and length of education. Our data on taxable income cover the years 1999–2010 and these are the years utilized in our mean annual income measure.

### Ethics statement

The research described in this paper was approved by the Ethics Committee in Stockholm, Sweden (Regionala Etikprövningsnämnden, Stockholm) where this project was handled as protocol 05/2011. The project was also approved by the Committee on the Use of Human Subjects/Institutional Review Board at Harvard University (where the project's reference number was #F–21619). When subjects received the survey, it was described in an accompanying letter that their answers would be linked to administrative data (it was specified which databases would be used) and that any identifiers would be removed by Statistics Sweden before the data would be made available to the researchers. Furthermore, the letter pointed out that a subject who sent back a filled-out survey would be regarded as having given his consent to the data being used in the described way. This way of obtaining consent was approved by both the Swedish Ethics Committee and the Harvard IRB.

## Supporting Information

File S1 **Supplementary file containing Tables S1–S3.** Table S1: Ordered probit. Table S2: Robustness regressions. Table S3: Controlling for age-group differences.
(DOC)

## References

[1] Deary IJ, Batty GD, Gale CR (2008) Childhood intelligence predicts voter turnout, voting preferences, and political involvement in adulthood: The 1970 British Cohort Study. Intelligence 36: 548–555.

[2] Judge T, Colbert A, Ilies R (2004) Intelligence and leadership: A quantitative review and test of theoretical propositions. J App Psych 89: 545–552. 10.1037/0021-9010.89.3.542 15161411

[3] Hodson G, Busseri M (2012) Bright minds and dark attitudes. Psych Sci 23(2): 187–195. 10.1177/0956797611421206 22222219

[4] Deary IJ, Batty GD, Gale CR (2008) Bright children become enlightened adults. Psych Science 19: 1–6. 10.1111/j.1467-9280.2008.02036.x 18181782

[5] Schoon I, Cheng G, Gales CR, Batty GD, Deary IJ (2010) Social status, cognitive ability, and educational attainment as predictors of liberal social status and political trust. Intelligence 38: 144–150.

[6] Keiller SW (2010) Abstract reasoning as a predictor of attitudes toward gay men. J Homosexual 57: 914–927. 10.1080/00918369.2010.493442 20665331

[7] Alesina A, DiTella R, MacCulloch R (2004) Inequality and happiness: are Europeans and Americans different? J Publ Econ 88: 2009–2042.

[8] Alesina A, Glaeser E (2004) Fighting poverty in the US and Europe: A world of difference. Oxford: Oxford University Press. 250p.

[9] Alesina A, Giuliano P (2011) Chapter 4 in Handbook of social economics vol 1B, Bisin A, Benhabib J, Jackson M, Eds. Amsterdam: North Holland.

[10] Fong C (2001) Social preferences, self-interest, and the demand for redistribution. J Public Econ 82: 225–246.

[11] Alesina A, La Ferrara E (2005) Preferences for redistribution in the land of opportunities. J Publ Econ 89: 897–931.

[12] Benabou R, Ok E (2001) Social mobility and the demand for redistribution: the POUM hypothesis. Q J Econ 116: 447–487.

[13] Romer T (1975) Individual welfare, majority voting and the properties of a linear income tax. J Publ Econ 7: 163–188.

[14] Meltzer A, Richards S (1981) A rational theory of the size of the government. J Pol Econ 89: 914–927.

[15] Fong C, Luttmer E (2011) Do fairness and race matter in generosity? Evidence from a nationally representative charity experiment. J Publ Econ 95: 372–394.

[16] Alesina A, Angeletos GM (2005) Fairness and Redistribution. Am Econ Rev 95(4): 960–980.

[17] Lindqvist E, Westman R (2011) The labor market returns to cognitive and noncognitive ability: evidence from the Swedish enlistment. Am Econ J: App Econ 3: 101–128.

[18] Heckman J, Sixrud J, Urzua S (2006) The effects of cognitive and noncognitive abilities on labor market outcomes and social behavior. J Labor Econ 24: 411–482.

[19] Benjamin D, Brown S, Shapiro J (2013) Who is ‘behavioral’? Cognitive ability and anomalous preferences. J Eu Econ Ass 11(6): 1231–1255. 10.1111/jeea.12055 PMC6289538 30546272

[20] Dohmen T, Falk A, Huffman D, Sunde U (2011) Are risk aversion and impatience related to cognitive ability? Am Econ Rev 100(3): 1238–1260 (2010)..

[21] Frederick S (2005) On the ball: Cognitive reflection and decision-making. J Econ Perspect 19(4): 25–42.

[22] Burks SV, Carpenter JP, Goette L, Rustichini A (2009) Cognitive skills affect economic preferences, strategic behavior, and job attachment. PNAS 106(19): 7745–7750. 19416865 10.1073/pnas.0812360106 PMC2683075

[23] Rustichini A, DeYoung C, Anderson J, Burks S (2012) Toward the integration of personality theory and decision theory in the explanation of economic and health behavior. IZA Discussion Paper 6750.

[24] DeYoung CG, Quilty LC, Peterson JB, Gray JR (2014) Openness to experience, intellect, and cognitive ability. J Pers Asses 96(1): 46–52. 10.1080/00223891.2013.806327 23795918

[25] Sobel ME (1982) Asymptotic confidence intervals for indirect effects in structural equation models. Sociol Methodol 13: 290–213.

[26] Sobel ME (1986) Some new results of indirect effects and their standard errors in covariance structure models. Sociol Methodol 16: 159–186.

[27] MacKinnon DP (2008) Introduction to statistical mediation analysis, London: Routledge.

[28] Carlstedt B (2000) Cognitive abilities: aspects of structure, process and measurement. Thesis, Gothenburg University, Sweden.

[29] Holt C, Laury S (2002) Riskaversion and incentive effects. Am Econ Rev 92: 1644–1655.

[30] Falk A, Becker A, Dohmen T, Huffman D, Sunde U (2013) An Experimentally validated preference survey module, University of Bonn, Germany.

