# Correction: Cognitive Ability and the Demand for Redistribution

**DOI:** 10.1371/journal.pone.0115484

**Published:** 2014-12-08

**Authors:** The PLOS ONE Staff

The following information is missing from the Funding section: Publication of this article was funded in part by the George Mason University Libraries Open Access Publishing Fund. Please refer to the complete funding statement here.

Financial support for this project is gratefully acknowledged from: the Ragnar Söderberg Foundation (http://ragnarsoderbergsstiftelse.se/, grant number: E56-10), Kungliga VetenskapsAkademin (http://www.kva.se/, grant number: FOA10H-172) and the Lab for Economics Applications and Policy at Harvard (http://leap.fas.harvard.edu/, grant number: 370 31890 017599 619701 0012 50963). Publication of this article was funded in part by the George Mason University Libraries Open Access Publishing Fund. The funders had no role in study design, data collection and analysis, decision to publish, or preparation of the manuscript.
