# Supplementary material for: Cognitive Ability and the Demand for Redistribution
Source: PLoS One. 2014 Oct 24;9(10):e109955. doi: 10.1371/journal.pone.0109955 (PMC4208759; doi:10.1371/journal.pone.0109955)
Supplement: Supplementary file 1 [file pone.0109955.s001.doc]

Table S1. Ordered Probit

	(1)	(2)	(3)	(4)	(5)	(6)	(7)	(8)	(9)	(10)	(11)	(12)	(13)	(14)	(15)	(16)
Cognitive ability	-0.263***	-0.205***		-0.179**		-0.176**		-0.216***		-0.186**		-0.198***		-3.696**		-3.475*
	(0.065)	(0.069)		(0.071)		(0.071)		(0.069)		(0.072)		(0.070)		(1.796)		(1.858)
Mean annual income			-0.009**	-0.006*									-0.007*	-0.138	-0.005	-0.101
			(0.004)	(0.004)									(0.004)	(0.087)	(0.004)	(0.095)
Beliefs: luck/effort					-0.119***	-0.108**							-0.109**	-2.403**	-0.100**	-2.146**
					(0.042)	(0.042)							(0.043)	(0.992)	(0.045)	(1.048)
Beliefs: gov efficiency							0.034**	0.037***							0.105***	2.183***
							(0.014)	(0.014)							(0.033)	(0.751)
Risk aversion									0.037	0.039					0.029	0.736
									(0.038)	(0.037)					(0.038)	(0.864)
Altruism											-0.151	0.003			-0.009	-0.005
											(0.723)	(0.029)			(0.029)	(0.680)
Standard controls	No	Yes	Yes	Yes	Yes	Yes	Yes	Yes	Yes	Yes	Yes	Yes	Yes	Yes	Yes	Yes
N	271	266	266	266	265	265	260	260	247	247	262	262	265	265	243	243

OP. Robust standard errors in parentheses. Significance levels: ***p<0.01, **p<0.05, *p<0.1. Dependent variable is demand for redistribution measured in percent where 0% = no redistribution, 100%=full redistribution. Cognitive ability measure from military enlistment data with zero mean and unit standard deviation in the population of all enlisters. Standard controls are age, education level (dummy for primary school being highest education level), and self-reported socio-economic status during childhood (alternatives "working class", "lower middle class", "middle class", "upper middle class" and "upper class"). Mean annual income is measured in SEK 10,000s (exchange rate at time of survey was 1 USD = 6.1 SEK), mean taken over the years 1999 to 2010. Beliefs about luck/effort measured with question in survey about what matters (luck or effort) for how well an individual does economically in life, higher number indicates more importance for effort. Beliefs about government efficiency measured in survey with question about how efficiently the public sector in Sweden redistributes resources so that no resources get lost on the way, higher number indicate more efficiency. Risk aversion measured in survey with eight hypothetical choices between a fixed amount and a lottery, risk aversion is sum of choices of the fixed amount. Altrusim assessed in survey with hypothetical question about the willingness to give to charitable purposes (1=willing to give, 0=not willing to give).

Table S2. Robustness regressions

	(1)	(2)	(3)	(4)	(5)	(6)	(7)	(8)
Cognitive ability	-3.631**	-4.003**	-4.051**	-3.985**	-4.031**	-4.153**	-4.721***	-4.721***
	(1.734)	(1.786)	(1.783)	(1.796)	(1.796)	(1.827)	(1.742)	(1.746)
Current income	-0.270**							
	(0.106)							
Current income sq	0.001**							
	(0.000)							
Current income positive	27.859***							
	(5.466)							
Mean income		-0.689***	-0.651**	-0.680***	-0.682***	-0.771***		
		(0.237)	(0.260)	(0.237)	(0.239)	(0.266)		
Mean income sq		0.005**	0.005**	0.005**	0.005**	0.005**		
		(0.002)	(0.002)	(0.002)	(0.002)	(0.002)		
Mean income positive		-2.591	-2.963	-2.827	-2.717	-11.986*		
		(7.060)	(7.178)	(7.126)	(7.101)	(6.635)		
Share wage income			-1.617					
			(6.457)					
Income variability				0.000				
				(0.000)				
Government transfers					0.001			
					(0.005)			
Beliefs about future 5 years						0.975		
						(2.367)		
Beliefs about future 10 years						-1.700		
						(2.087)		
Wealth							-0.009**	-0.009**
							(0.003)	(0.004)
Wealth sq							0.000***	0.000***
							(0.000)	(0.000)
Wealth positive							7.088*	7.100*
							(3.862)	(4.006)
Share financial wealth								-0.006
								(0.184)
Standard controls	Yes	Yes	Yes	Yes	Yes	Yes	Yes	Yes
N	266	266	266	266	266	262	266	266

OLS. Robust standard errors in parentheses. Significance levels: ***p<0.01, **p<0.05, *p<0.1. Dependent variable is Demand for redistribution measured in percent where 0% = no redistribution, 100%=full redistribution. Cognitive ability measure from military enlistment data with zero mean and unit standard deviation in the population of all enlisters. Standard controls are age, education level (dummy for primary school being highest education level), and self-reported socio-economic status during childhood (alternatives "working class", "lower middle class", "middle class", "upper middle class" and "upper class"). Current income is measured in SEK 10,000s in 2010. Mean annual income is measured in SEK 10,000s, mean taken over the years 1999 to 2010. "Sq" indicates that the square of the variable is included. "Positive" indicates that a dummy for whether the income measure is positive is included. "Share wage income" is share of mean income from wage. "Income variability" is standard deviation of mean income 1999-2010. Government transfers are unemployment compensation and compensation for sick leave. Beliefs about future income is self-reported beliefs about relative income in 5 and 10 years (scale 1-10 where higher number indicate higher relative income). Wealth measured in SEK 10,000s. "Share financial wealth" indicates share of total wealth in financial assets.

Table S3. Controlling for age-group differences

	(1)
Cognitive ability	-5.159
	(3.440)
Cogn diff age 33-42	0.877
	(4.415)
Cogn diff age 43-53	2.175
	(4.058)
Mean income	-0.677***
	(0.242)
Mean income sq	0.005**
	(0.002)
Positive mean income	-2.163
	(8.877)
Standard controls	Yes
N	266

Significance levels: ***p<0.01, **p<0.05, *p<0.1. Cogn Diff Age 33-42 and Cogn Diff Age 43-53 indicate how the coefficient on cognitive ability differs for these age groups from the coefficient for age group 54-61. All other variables defined as in Table S2.
